# Association between internet use and depression among the middle-aged and elderly adults with multimorbidity in China: do gender differences exist?

**DOI:** 10.3389/fpsyt.2024.1494979

**Published:** 2025-01-20

**Authors:** Jiao Zhang, Yuheng Jia, Lixin Hong, Yixin Zhang, Lihua Li, Kan Tian

**Affiliations:** ^1^ School of Health Economics and Management, Nanjing University of Chinese Medicine, Nanjing, China; ^2^ Jiangsu Research Center for Major Health Risk Management and TCM Control Policy, Nanjing University of Chinese Medicine, Nanjing, China; ^3^ School of Pharmacy, Nanjing University of Chinese Medicine, Nanjing, China; ^4^ School of Elderly Care Services and Management, Nanjing University of Chinese Medicine, Nanjing, China

**Keywords:** internet use, depression, aging, multimorbidity, gender difference

## Abstract

**Objectives:**

Internet use and the results of mental health are related. Numbers of studies presented the association between Internet use and depression, and the middle-aged and elderly adults with multimorbidity are of concern. The study aimed to explore the relationship between Internet use and depression in middle-aged and elderly adults with multimorbidity.

**Methods:**

We selected 2550 respondents aged 45 years and above with multimorbidity from the China Health and Retirement Longitudinal Study (CHARLS) 2018 database. Logistic regression models were constructed to examine the effects of Internet use on depression, as well as comparing gender differences. Meanwhile, propensity score matching (PSM) was used to test the robustness of the results.

**Results:**

Overall, 49.8% of respondents had a risk of developing depression, and 14.9% of the participants used the Internet. Internet use (*OR* = 0.66, *P* = 0.002), type of devices (one type: *OR* = 0.69, *P* = .011;≥2 types: *OR* = 0.53, *P* = 0.03), frequency of Internet use (regularly: *OR* = 0.67, *P* = 0.005) were all inversely associated with depression. Significant differences between genders were observed, Internet use was associated with a lower prevalence of depression among men, while the association was not statistically significant among women.

**Conclusions:**

There is a significantly negative association between Internet use and depression in the middle-aged and elderly adults with multimorbidity in China, and this relationship varies across different genders. This suggests that Internet use may be a protective factor for depressive symptoms in the older population, offering a guideline for policymakers to develop specific strategies for different genders.

## 1 Introduction

Ageing is a rapidly increasing trend worldwide. From 2020 to 2030, the proportion of the global population aged 60 years and up is estimated to increase by 34% (1). China has the largest elderly population in the world, with 264 million elderly people aged 60 and above in 2020, accounting for 18.7% of the total population (2). There is no doubt that health is the issue of greatest concern in the aging process. As age increases, organs begin to deteriorate and functional recession intensifies, making the older populations increasingly vulnerable to disease risks. As such, morbidity and mortality due to chronic diseases mainly occur in older individuals (3). The coexistence of 2 or more chronic diseases is defined as multimorbidity (4). On account of the extension of life expectancy globally, multimorbidity becomes progressively more common with age, and places a profound burden both physically and mentally (5, 6). Therefore, it is necessary for us to concentrate on the middle-aged and elderly adults with multimorbidity.

Depression is an affective disorder characterized by a persistent sensation of negativity, accompanied by functional deterioration in organs (7). Depression is a common global health priority that widely distributed in the population, posing huge challenges to the health care system (8). The projections of the World Health Organization suggested that it will be the most common disabling disease around the world by 2030 (9). As one of the most prevalent psychiatric disorders diminishing the elderly people’s quality of life, it has become an urgent and growing concern globally (10). Chronic disease is a risk factor for depressive symptoms for the elderly (11). Also, risk of depression is doubled with multimorbidity compared to those with one chronic disease (12, 13). Consequently, improving the depression status in middle-aged and elderly adults with multimorbidity needs to be focused on.

Currently, the Internet is increasingly becoming an integral part of everyday life. The Internet is an important tool that provides entertainment, living necessities and health-related information (14). The Internet penetration rate in China has reached 77.5%, and the proportion of netizen aged 50 and above was 32.5%, which is growing markedly and continuously (15). The association between Internet use and depression was well established, and consequences were mainly divided into two directions. The majority suggested that the Internet was a protective factor for depressive symptoms (16, 17). Meanwhile, some studies drew contrary conclusions that overuse of Internet could promote depression (18, 19). Few studies have examined the association from the perspective of the population with multimorbidity. Due to possible technical barriers and physical inconvenience for older adults with multimorbidity (20), the relationship between Internet use and mental health outcomes still remains unclear. In addition, gender is associated with late-life depression. Owing to the gender difference in physiological and socioeconomic aspects, depression affects men and women apart (21). Previous studies shown that the prevalence of depressive symptoms was significantly higher among women (22, 23). Consequently, it is crucial to take gender disparities into account in exploring the connection between Internet use and depression. Therefore, the purpose of this study is to clarify the association between Internet use and depression among the middle-aged and elderly adults with multimorbidity, and to further explore whether there are gender differences in the association.

## 2 Methods

### 2.1 Study design and participants

We used the publicly available 2018 CHARLS data (the China Health and Retirement Longitudinal Study, wave 4) for cross-sectional analysis, which was released in September 2020. CHARLS was conducted by the Institute of Social Science Survey at Peking University, which aims to provide a series of high-quality data source for the current study on aging population. This longitudinal study features large-scale as well as highly representative, involving 19816 residents from 150 counties/districts and 450 urban communities/villages. It offers an overall analysis of the Chinese older individuals, including their household, demographic features, physical health, mental health, and economic status (24).

**Figure 1:**
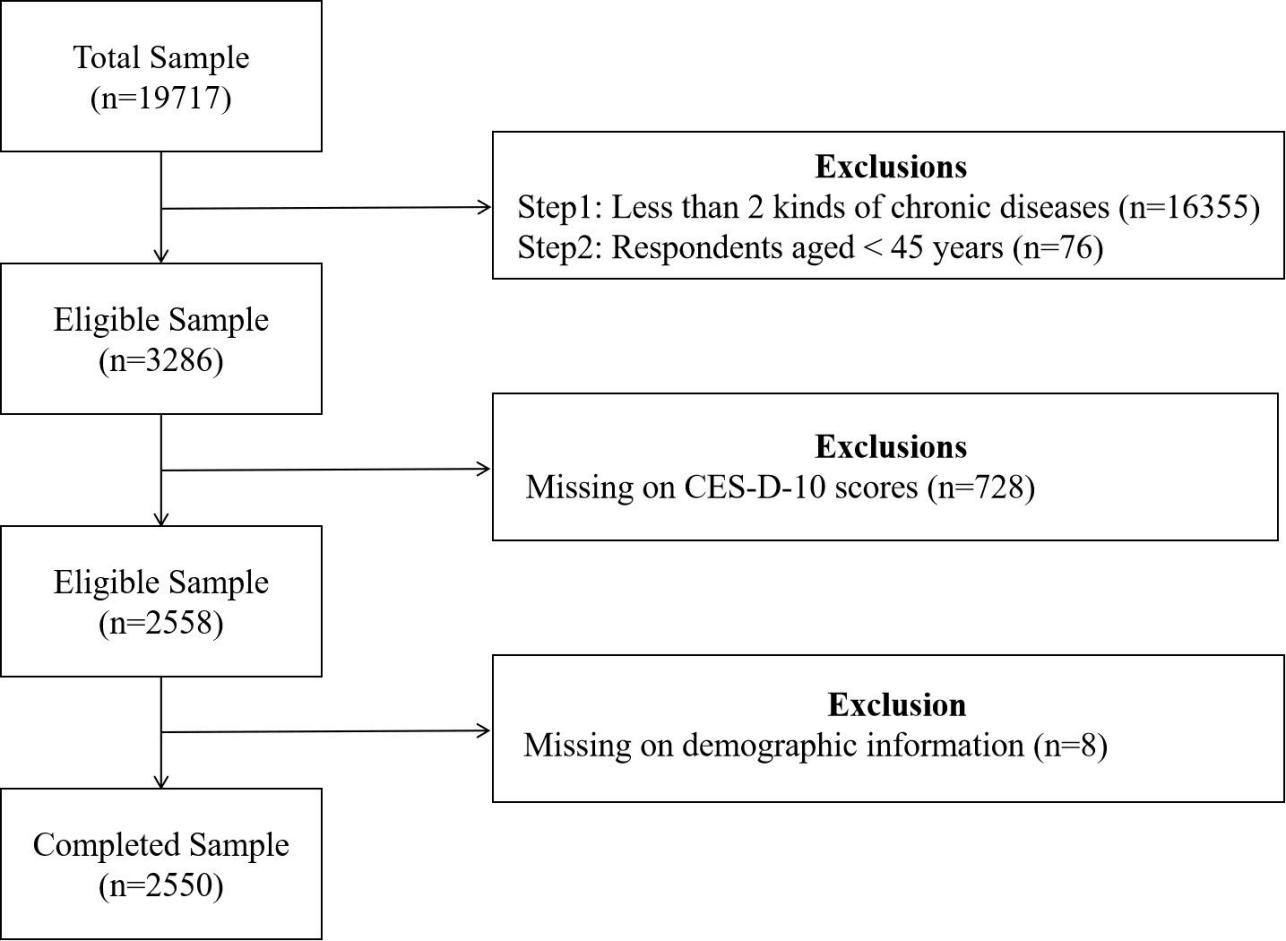
Flowchart of participant selection.

In our study, multimorbidity covered fourteen noncommunicable kinds of chronic diseases: hypertension, dyslipidemia, diabetes, cancer (excluding minor skin cancers), chronic lung diseases, liver diseases (except fatty liver and tumors), heart diseases (including heart attack, coronary heart disease, angina, congestive heart failure, or other heart problems), stroke, kidney diseases (except tumors), digestive diseases (except tumors), emotional and psychiatric diseases, memory-related disorders, arthritis or rheumatism and asthma. According to the research purpose, we selected people 45 years and above with multiple chronic diseases as the participants. Finally, a total of 2550 participants were enrolled, from which the following sample data were omitted: (i) have been diagnosed by a doctor with less than 2 kinds of chronic diseases; (ii) age <45 years; (iii) missing data of CES-D-10 scores; (iv) missing data of demographic factors. The participant selection process is displayed in **Figure 1**.

### 2.2 Variables

#### 2.2.1 Independent variable

The main independent variable was Internet use. Based on the question “Have you done any of these activities in the last month?” in the CHARLS 2018 household questionnaire, we intercepted one of the options “used the Internet” (Yes, No) to construct our main independent variable.

Type of devices and frequency of Internet use were also independent variables. According to the questions “Which types of devices do you use to access the Internet?” “How often in the last month did you use the Internet?”, “type of devices” (0/1/≥2) and “frequency of Internet use”: Never/Not regularly/Regularly (≥once a week: including almost daily and almost every week) were constructed respectively. Types of devices referred to desktop computer, laptop computer, tablet computer, cellphone and other devices.

#### 2.2.2 Dependent variables

By virtue of the Center for Epidemiology Studies Depression Scale (CES-D-10) in the CHARLS questionnaire, we measured the depressive symptoms and considered it as the dependent variable. The CES-D-10 is a reliable tool for epidemiological survey, which was developed by Andresen in 1977. The scale contains 10 different items regarding the respondents’ behaviors during the previous week, such as feeling fretful, difficulty in concentrating, feeling depressed, feeling fearful and vision for the future. Each item is divided into 4 levels (0 = none or rarely; 1 = for a little while; 2 = occasionally or half of the time; 3 = most of the time) and the total score ranges from 0 to 30. Higher scores indicate more severe depressive symptoms (25). Referring to former studies, the depression variable was ultimately constructed with a cut-off point of 10. A total score of ≥10 was defined as depressive symptoms, a score of <10 was defined as normal (26, 27). Moreover, the scale shows good internal consistency (Cronbach’s alpha = .820).

#### 2.2.3 Control variables

Two types of control variables were selected, including demographic characteristics and lifestyle factors. The former embodied gender (man, woman), age (45-50, 51-60, 61-70, >70), residence (urban, rural), education (illiterate, primary school, middle school, high school, college and above) and marital status (married, unmarried), the latter included sleep duration (<6h, 6-8h, >8h), tobacco use (yes, no), alcohol use (yes, no), physical activities (yes, no), which were all dichotomous variables. We defined “married and living with spouse present” and “married but not living with spouse temporarily” as married; correspondingly, “separated” “divorced” “widowed” and “never married” constituted unmarried. Physical activities comprised any act performed for at least 10 minutes every time over the course of a week, such as carrying heavy stuff, hoeing, mopping, aerobic workout, speed walking and so on.

### 2.3 Statistical analysis

Using StataMP 17.0 for preliminary data process and further analysis. Given that all variables were categorical variables, the Chi-square test was used to compare the association between various types of variables and depression. To identify the association between Internet use and depression as well as analyzing gender differences, univariate and multivariate logistic regression analysis were successively used. Model 1-2 examined the relationship between Internet use and depression. Model 3 analyzed the association between type of devices and depression. Model 4 analyzed the association between frequency of Internet use and depression. Model 5-6 performed subgroup analysis of chronic diseases, for groups with two chronic diseases and groups with three or more chronic diseases respectively. Model 1 conducted univariate analysis; Model 2-6 contained all control variables. Also, gender differences were compared in the following model. The difference was considered statistically significant if *P*-value <.05. In addition, to reduce the selectivity bias, the propensity score matching method (PSM) was applied for robustness testing.

## 3 Results

### 3.1 Characteristic of participants according to depressive symptoms

**Table 1** depicts the basic characteristics in the whole sample. 2550 middle-aged and elderly adults with multimorbidity were included. The mean age of all the participants was 61.96 years, the mean age of Internet users and non-users was respectively 57.07 years and 62.83 years. Of all the participants, a greater proportion were women (52.0%) and married (85.8%). Additionally, majority of participants participated in physical activities (90.6%). Overall, taking the total sample as a reference, 49.8% (1270/2550) of the participants had depressive symptoms. In all the individuals with depressive symptoms, being subclinical depressed was more prevalent among women (59.2%), rural residents (82.7%), married (82.8%), those did not smoke (62.8%), those did not consume alcohol (73.8%) and participants not using the Internet (90.2%). Among Internet users, participants using one type of devices (190/380, 50.0%) and those with regular Internet usage frequency (237/380, 62.4%) occupied the highest percentage and were also associated with no symptoms of depression.

**Table 1:** Basic characteristics of the participants (n=2550).

Variables	Total number	Depressive symptoms		χ^2^	C^b^
	N (%)	Yes	No		
Gender				52.16**	0.142**
Man	1223(47.96)	518(40.79)	705(55.08)		
Woman	1327(52.04)	752(59.21)	575(44.92)		
Age(year)				7.04	0.053
45-50	244(9.57)	107(8.43)	137(10.70)		
51-60	790(30.98)	401(31.57)	389(30.39)		
61-70	978(38.35)	475(37.40)	503(39.30)		
>70	538(21.10)	287(22.60)	251(19.61)		
Residence				46.92**	0.134**
Urban	588(23.06)	220(17.32)	368(28.75)		
Rural	1962(76.94)	1050(82.68)	912(71.25)		
Education				113.01**	0.206**
Illiterate	487(19.10)	323(25.43)	164(12.81)		
Primary school	1106(43.37)	583(45.91)	523(40.86)		
Middle school	590(23.14)	233(18.35)	357(27.89)		
High school	303(11.88)	113(8.90)	190(14.84)		
College and above	64(2.51)	18(1.42)	46(3.59)		
Marital status				18.84**	0.086**
Married	2189(85.84)	1052(82.83)	1137(88.83)		
Unmarried	361(14.16)	218(17.17)	143(11.17)		
Sleep duration(h)^a^				142.09**	0.230**
<6	1046(41.02)	668(52.60)	378(29.53)		
6-8	1309(51.33)	515(40.55)	794(62.03)		
>8	195(7.65)	87(6.85)	108(8.44)		
Tobacco use				23.11**	0.095**
No	1480(58.04)	797(62.76)	683(53.36)		
Yes	1070(41.96)	473(37.24)	597(46.64)		
Alcohol use				28.53**	0.105**
No	1756(68.86)	937(73.78)	819(63.98)		
Yes	794(31.14)	333(26.22)	461(36.02)		
Physical activities				15.39**	0.077**
No	241(9.45)	149(11.73)	92(7.19)		
Yes	2309(90.55)	1121(88.27)	1188(92.81)		
Internet use				51.07**	0.140**
No	2170(85.10)	1145(90.16)	1025(80.08)		
Yes	380(14.90)	125(9.84)	255(19.92)		
Type of devices				54.27**	0.144**
0	2170(85.10)	1145(90.16)	1025(80.08)		
1	294(11.53)	104(8.19)	190(14.84)		
≥2	86(3.37)	21(1.65)	65(5.08)		
Frequency of Internet use				51.07**	0.140**
Never	2170(85.10)	1145(90.16)	1025(80.08)		
Not regularly	27(1.06)	9(0.71)	18(1.41)		
Regularly	353(13.84)	116(9.13)	237(18.52)		

N.B. Total percentages of some variables are not equal to 100 due to rounding.

a : actual hours of sleep participants got at night (average hours for one night) during the past month.

b : coefficient of contingency

**: P <.01.

**Figure 2:**
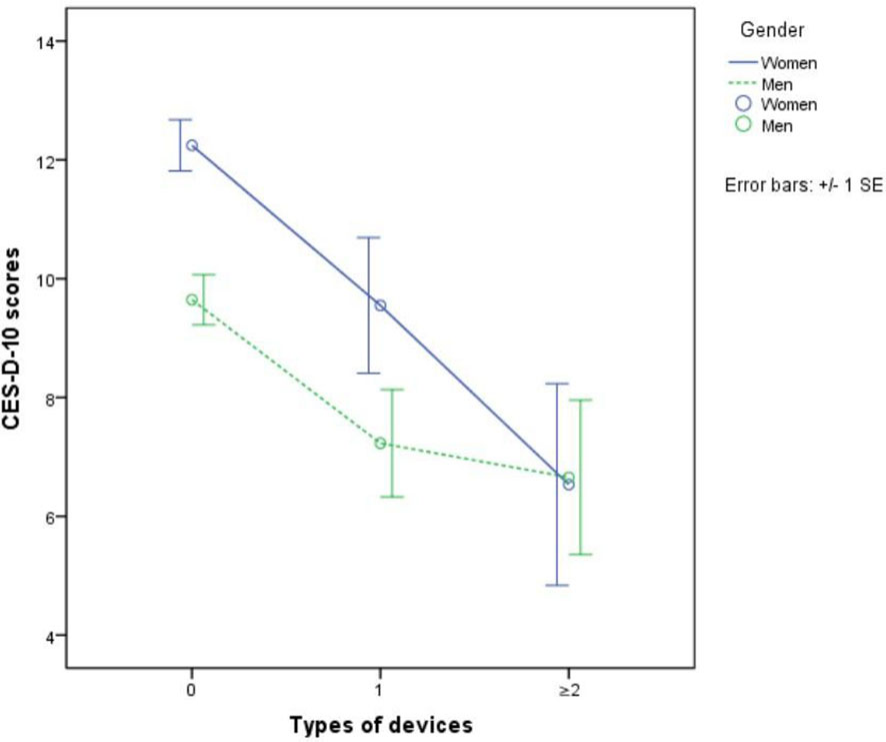
Mean CES-D-10 scores between types of devices by gender.

The mean CES-D-10 score of all participants was 10.57. In terms of Internet use, the mean CES-D-10 score was 7.96 in Internet users vs. 11.03 in Internet non-users. **Figure 2** demonstrates a downward trend in depression scores as the number of Internet-enabled devices increases. Among Internet users, men had lower mean CES-D-10 scores than women. The mean CES-D-10 score was lowest in both men and women who using two or more types of devices.

### 3.2 Comparison of type and frequency of internet use between men and women

**Table 2** summarizes participants’ Internet use by gender. Totally, 14.9% (380/2550) of the participants used the Internet. Among those who used Internet, the majority used one type of device (11.5%), with a frequency of more than once a week (13.8%). The proportion of men using the Internet (17.2%) was higher than that of women (12.8%), with more types and higher frequency (15.6% men vs 12.2% women used the Internet with a regular frequency) of Internet use.

**Table 2:** Comparison of type and frequency of Internet use in men and women.

Variables	Total sample	Men	Women	χ^2^	P
	N (%)	N (%)	N (%)		
Internet use				9.54	.002
No	2170(85.10)	1013(82.83)	1157(87.19)		
Yes	380(14.90)	210(17.17)	170(12.81)		
Type of devices				16.15	<.001
0	2170(85.10)	1013(82.83)	1157(87.19)		
1	294(11.53)	152(12.43)	142(10.70)		
≥2	86(3.37)	58(4.74)	28(2.11)		
Frequency of Internet use				12.20	.002
Never	2170(85.10)	1013(82.83)	1157(87.19)		
Not regularly	27(1.06)	19(1.55)	8(0.60)		
Regularly	353(13.84)	191(15.62)	162(12.21)		

N.B. Total percentages of some variables are not equal to 100 due to rounding.

### 3.3 Association between internet use and depression

Regression results for the relationship between Internet use and depression are shown in **Table 3**. Model 1 only measured Internet use and depression. Based on the results of the coefficient of contingency test, we found that all variables except age were significantly associated with depressive symptoms. Since age is a common underlying demographic variable, we included it in the analysis. Consequently, we added all control variables to Model 2-6. From all the models, we could find that Internet use was consistently and significantly associated with a lower likelihood of subclinical depression.

**Table 3:** Logistic regression results of the association between Internet use and depression (n=2550).

Variables	Model 1	Model 2	Model 3	Model 4	Model 5	Model 6
	OR^a^ (95%CI)	OR^b^ (95%CI)	OR^b^ (95%CI)	OR^b^ (95%CI)	OR^b^ (95%CI)	OR^b^ (95%CI)
Internet use						
No	Ref.	Ref.			Ref.	Ref.
Yes	0.44**(0.35,0.55)	0.66**(0.51,0.86)			0.62**(0.44,0.87)	0.71(0.47,1.08)
Type of devices						
0			Ref.		Ref.	Ref.
1			0.69*(0.52,0.93)		0.62*(0.42,0.90)	0.77(0.50,1.20)
≥2			0.53*(0.31,0.92)		0.61(0.31,1.23)	0.45(0.18,1.12)
Frequency of Internet use						
Never				Ref.	Ref.	Ref.
Not regularly				0.54(0.23,1.24)	0.52(0.17,1.59)	0.57(0.16,2.06)
Regularly				0.67**(0.51,0.89)	0.63*(0.44,0.90)	0.73(0.47,1.12)

Model 1-Model 4: Analysis for total sample;

Model 5: Analysis for the sample with two chronic diseases;

Model 6: Analysis for the sample with three or more chronic diseases;

a : OR was unadjusted;

b : ORs were adjusted for gender, age, residence, education, marital status, sleep duration, tobacco use, alcohol use, physical activities.

*: P <.05,

**: P <.01.

Upon adjusting for additional variables that could cause confusion, Internet use was significantly and negatively associated with depression (Model 2: *OR* = 0.66, 95% *CI*: 0.51-0.86, *P* = .002). Type of devices was also significantly associated with a lower possibility of subclinical depression (Model 3: one type: *OR* = 0.69, 95% *CI*: 0.52-0.92, *P* = .011;≥2 types: *OR* = 0.53, 95% *CI*: 0.31-0.92, *P* = .03). Moreover, those who using the Internet at a regular frequency was less likely to get depressed (Model 4: *OR* = 0.67, 95% *CI*: 0.51-0.89, *P* = .005).

We further performed a subgroup analysis for the population with different kinds of chronic diseases. As for the population with two chronic diseases, those who were Internet users (Model 5: *OR* = 0.62, 95% *CI*: 0.44-0.87, *P* = .006), using one type of devices (Model 5: *OR* = 0.62, 95% *CI*: 0.42-0.90, *P* = .012) and with regular Internet usage frequency (Model 5: *OR* = 0.63, 95% *CI*: 0.44-0.90, *P* = .01) were much less likely to suffer from depression. For the group with three or more chronic diseases, no significant association was found.

**Table 4** examined gender differences in the relationship between Internet use and depression. Significant gender disparity was observed among Internet users. Internet use (yes: *OR* = 0.58, 95% *CI*: 0.40-0.85, *P* = .004), type of devices (one type: *OR* = 0.57, 95% *CI*: 0.37-0.86, *P* = .008) and frequency of Internet use (regularly: *OR* = 0.58, 95% *CI*: 0.39-0.85, *P* = .006) were significantly associated with lower likelihood of depression in men. As for women, no significant associations were found between different dimensions of Internet use and depression.

**Table 4:** Gender differences in the association between Internet use and depression.

Variable	Men		Women	
	OR (95%CI)		OR (95%CI)	
Internet use		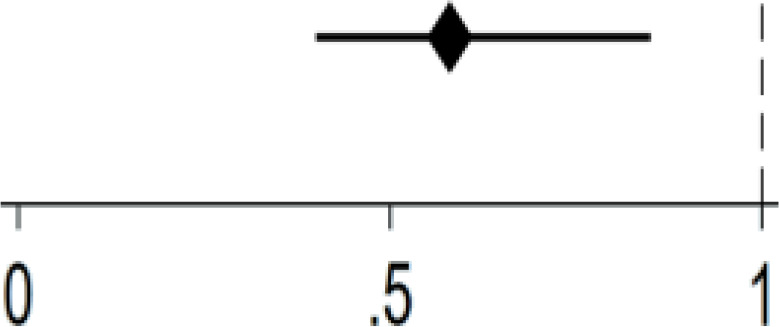		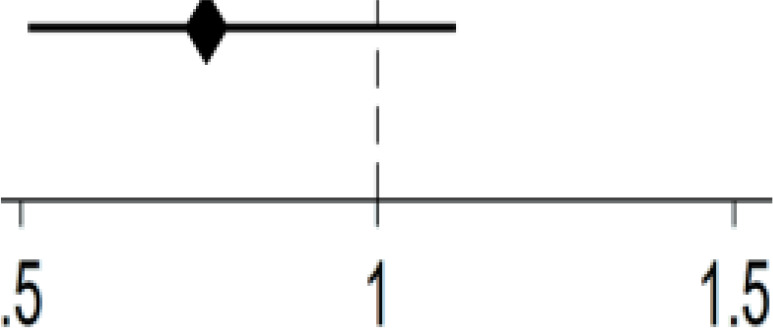
Yes	0.58**(0.40,0.85)		0.76(0.51,1.11)	
No	Ref.		Ref.	
Type of devices		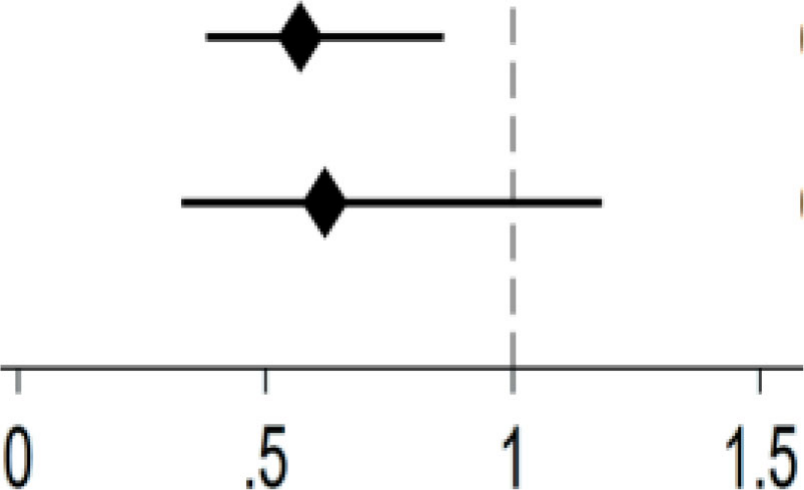		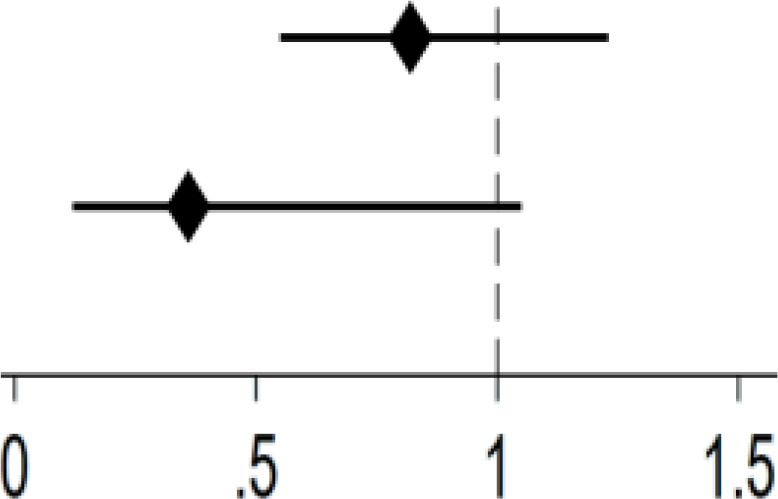
1	0.57**(0.38,0.86)		0.82(0.55,1.23)	
≥2	0.62(0.33,1.18)		0.36(0.12,1.05)	
0	Ref.		Ref.	
Frequency of Internet use		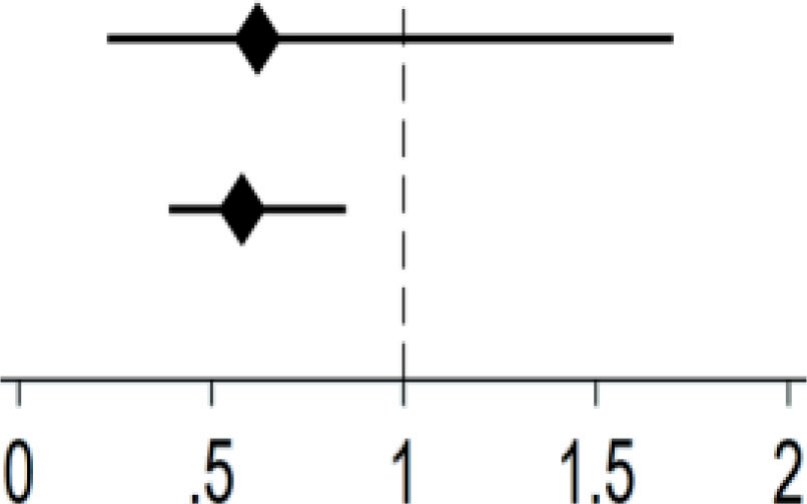		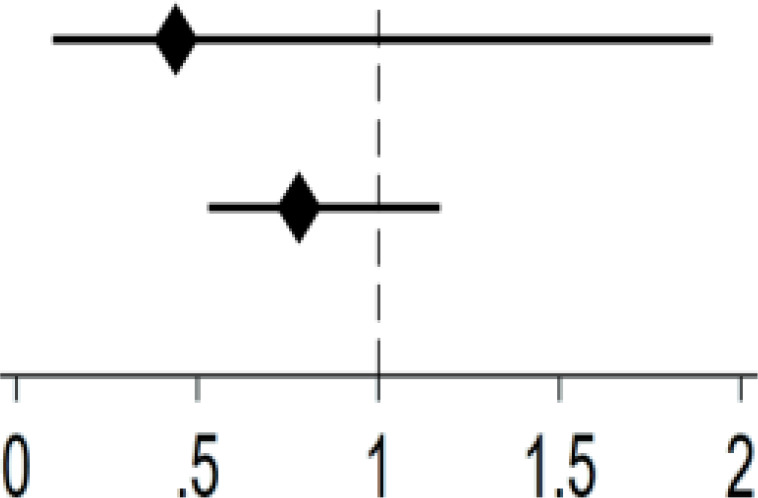
Not regularly	0.62(0.23,1.70)		0.44(0.10,1.92)	
Regularly	0.58**(0.39,0.85)		0.78(0.53,1.17)	
Never	Ref.		Ref.	

N.B. ORs were adjusted for age, residence, education, marital status, sleep duration, tobacco use, alcohol use, physical activities;

**: P<.01.

### 3.4 Robustness test

**Figure 3:**
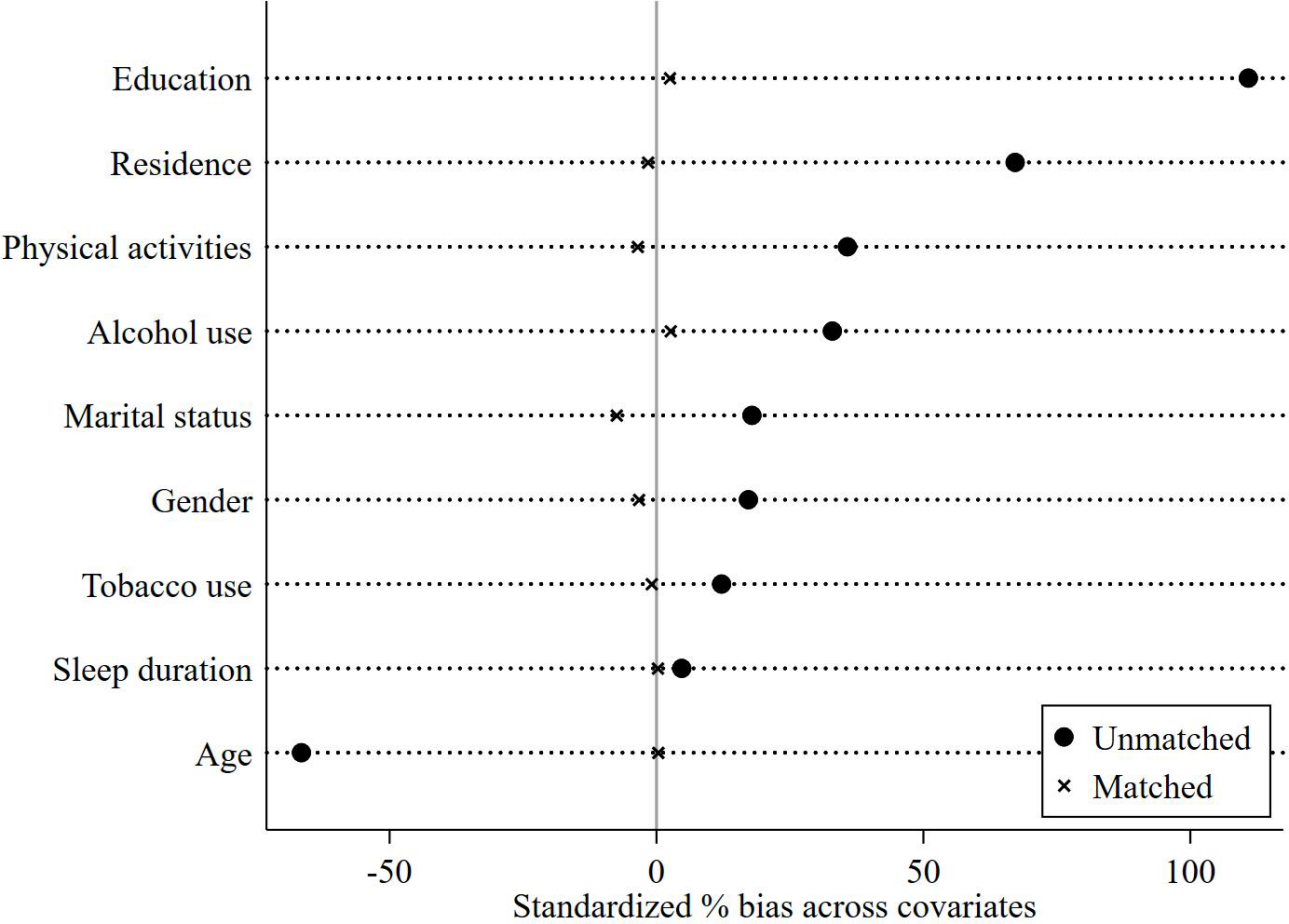
PSM balance test results.

**Figure 3** presents the results of the balance test. The results indicate the difference between the treated and control groups before and after matching as a way of determining the effect of matching. Before matching, the target population differed greatly in individual and social characteristics, while the absolute value of standardized errors after sample matching was less than 5%, indicating that it can be assumed that there is no difference between the treated and control groups. Nearest neighbor matching, radius matching and kernel matching methods were used to examine the average treatment effect (ATT) of Internet use on depression of middle-aged and older adults, and the ATT values estimated by the three methods were approximate, and the t-values were all significant at the 5% level, which indicated that the results of this study were robust (**Table 5**). After mitigating the effect of confounding factors, the effect of Internet use on the mental health status of middle-aged and older adults remained significant.

**Table 5:** PSM results of the effect of Internet use on depression.

Method	Sample	Treated	Controls	ATT	T-stat
/	Unmatched	0.329	0.528	−0.199	−7.22
Nearest neighbor matching	Matched	0.329	0.425	−0.096**	−2.57
Radius matching	Matched	0.329	0.455	−0.127**	−3.77
Kernel matching	Matched	0.329	0.448	−0.119**	−3.82

**: P<.01.

## 4 Discussion

Using cross-sectional data from a nationally representative sample, our study presented an insight into the effect of Internet use on depression among the middle-aged and elderly adults with multimorbidity. In this study, we produced three main findings. Firstly, the prevalence of depression among middle-aged and elderly adults with multimorbidity in China was particularly high, and the proportion of Internet users was low. Secondly, Internet use was generally associated with the lower likelihood of depression after controlling for other confounding variables. Thirdly, Internet use, type of devices and frequency of Internet use had a more significant association with depression among men, compared with women.

Referring to the overall middle-aged and elderly adults in China, our target with multimorbidity had a much higher prevalence of depressive symptoms (49.8% vs. 33.5%) (28). Moreover, the prevalence of depressive symptoms in the aging population significantly varied due to gender, and in which women occupied a higher rate, which was in line with other researches (29). As a consequence, the depressive symptoms in middle-aged and elderly adults with multimorbidity must be identified and treated promptly, especially for women who are more vulnerable to the risk of depression.

The middle-aged and elderly adults with multimorbidity had a relatively limited majority of Internet use. This result is similar with previous findings (30, 31). Majority of those with multimorbidity used the Internet at a regular frequency, which is inconsistent with previous studies concentrating on the middle-aged and elderly adults (17). Since middle-aged and elderly adults with multimorbidity tend to seek more health-related information, they use the Internet more frequently (32). With respect to the frequency of Internet use and types of devices, men were more active than women, and the number of using two types and more of devices for the Internet among females was especially limited. This is probably due to unequal gender division of labor, women were more involved in household labor (33). Women are taking on more hours of unpaid work than men, consisting mainly of household chores and parenting (34). Consequently, women perform a relative lack of spare time to use the Internet. According to several researches, since different genders use the Internet for different purposes, men are further vulnerable to problematic Internet use (35). As a result, sensible use of the Internet is worth promoting.

Previous studies have examined the association between Internet use and depression, and our finding is consistent with the negative result (16, 31). We found that for the elderly with multimorbidity, the Internet was inversely associated with depression. Additionally, further analysis revealed that this finding applies only to the group with two chronic diseases. In the elderly, depression mainly affects those with chronic diseases (36). Besides, for the middle-aged and elderly with multimorbidity, groups with two chronic diseases are the most numerous (37). Previous studies proposed two possible mechanisms by which Internet use reduces depression. Firstly, Internet can offer health-related information and more treatment options (38). People are capable of seeking relevant health information via the Internet, including prevention and management of depression (39). Also, Internet enables older persons to detect and treat depressive symptoms on time (40). Through mood tracking and clinical symptoms monitoring, smartphone apps are helpful to reduce depressive symptoms (41). Secondly, Internet could improve mental health by reducing social isolation. As one gets older, their social networks usually become narrow (42). Internet compensates for spatial barriers, enabling the seniors to reshape social networks (43).

Regarding frequency of Internet use, our study displayed that using the Internet regularly was associated with lower likelihood of depression. This finding behaves incoherently with previous studies concentrating on the youth, which indicates that more time spent on the Internet was related to poorer mental health (44). This could be because our target mostly had a limited frequency of Internet use, compared with teenagers who used the Internet almost every day. Moderate use of Internet may be worth taking into consideration. However, older adults may be trapped in the digital divide and have more technical barriers to Internet access (45). As society develops, the aged might be more disadvantaged in mastering the essential knowledge and skills than youthful ones (46). It is therefore important for the government to give priority to the relatively disadvantaged population, enhance the intelligence and convenience of electronic devices, and further increase the penetration of the Internet (47). As for types of devices, we found that using one type of device or using two types and more of devices for the Internet were both beneficial to reduce subclinical depression. The verdict contradicts other studies revealing that no association was found between types of devices to use the Internet and depression (17). The reason could be that getting online is more necessary for the population with restricted mobility. For a portion of seniors with multimorbidity and restricted mobility, Internet plays a more necessary role in enhancing social connection (43).

Additionally, compared with women, the results of this study presented that Internet use was significantly associated with good mental health for men. This finding contradicts previous studies concentrating on the overall middle-aged and elderly adults, which showed that Internet use is particularly effective for the mental health of women (48). This finding highlights the specificity of the population with multimorbidity. Differences in patterns of multimorbidity between genders, and the association between patterns of multimorbidity and depression also varies by gender (49). Compared to women, men with more chronic diseases have higher risk of depression (50). Hence, it is necessary to deepen the reform to address the inequality across different genders and concentrate on the chronic disease cohort.

Using nationally representative data, our study suggested a cross-sectional association between Internet use and depression among the middle-aged and elderly adults with multimorbidity, and the results are reasonably well extrapolated and of high quality. Despite the strengths of this study, some limitations also existed. Firstly, this was a cross-sectional study, and we were not capable of exploring the causal relationship between Internet use and depression. Secondly, in the process of selecting control variables, we did not cover all factors that affect depression, but only considered the more influential ones. Then, we applied CES-D-10 to assess depression symptoms, but it cannot represent the reality of depression diagnosis. In the future, we plan to conduct further research through mechanistic exploration. Finally, we focused our study population on the Internet-users. However, the majority of middle-aged and older adults facing digital barriers nationwide have not received enough attention, and we intend to explore this group in more depth in future studies.

## 5 Conclusion

In accordance with our findings, there is a significantly negative association between Internet use and depression in Chinese middle-aged and elderly adults with multimorbidity, and this relationship varies across different genders.

This suggests that Internet access may be crucial for easing depressed symptoms in the older population, offering a guideline for policymakers and relevant activists acting to focus on and improve their mental health, especially for the population with multimorbidity. Given the differences in multimorbidity patterns and likelihood of developing subclinical depression between different genders, specific strategies should be developed for different population characteristics. This study may be helpful to promote new prevention strategies and treatment options for late-life depression, as well as a relatively lower risk of depression in the elderly.

## Data Availability

The original contributions presented in the study are included in the article/**Supplementary Material**. Further inquiries can be directed to the corresponding author.
